# Lysine Carboxymethyl Cysteinate, as a Topical Glutathione Precursor, Protects Against Oxidative Stress and UVB Radiation-Induced Skin Damage

**DOI:** 10.3390/antiox14050606

**Published:** 2025-05-17

**Authors:** Ping Gao, Xue Xiao, Xiao Cui, Hong Zhang, Xuelan Gu

**Affiliations:** Unilever R&D Shanghai, 66 Lin Xin Road, Shanghai 202305, China

**Keywords:** glutathione precursor, oxidative stress, UV protection, skin brightening, inflammation, lysine carboxymethyl cysteinate, spatial metabolomics

## Abstract

Lysine carboxymethyl cysteinate (LCC) is a synthetic substance obtained via lysine salification of S-carboxymethyl-cysteine. LCC has emerged as a promising glutathione (GSH) precursor. In this study, we sought to determine whether LCC could boost GSH levels and protect skin against oxidative stress. Experiments utilizing primary human keratinocytes and skin tissue samples revealed that LCC significantly increased endogenous GSH levels. LCC was able to pass through the stratum corneum and reach deep into the epidermis, where it enhanced the production of key metabolites involved in GSH biosynthesis. Then, the efficacy of LCC on skin protection was explored. LCC demonstrated protective effects by shielding keratinocytes from blue-light-induced oxidative stress and preventing ultraviolet B (UVB)-induced barrier disruption and pigmentation in a pigmented living skin equivalent (pLSE) model. In addition to its antioxidant properties, LCC also reduced the production of inflammatory mediators. Together, these findings underscore the multifaceted role of LCC in bolstering the natural antioxidant defenses of skin and preventing the accumulation of irreversible damage from the environment, thereby positioning it as a promising candidate for advancing skin health.

## 1. Introduction

Oxidative stress, characterized by an imbalance between the production of reactive oxygen species (ROS) and the ability of biological systems to detoxify these reactive intermediates [1], is a critical factor contributing to skin conditions including hyperpigmentation, wrinkling and acne vulgaris [2,3,4], and to the pathogenesis of skin pigmentary disorders such as melasma [5,6]. Importantly, skin endogenous cytoprotective mechanisms cope daily with exogenous stressors, of which ultraviolet radiation (UVR) is the most important. Increased oxidative stress is among the most significant factors underlying UV-induced skin damage. UV exposure, especially to UVA and UVB, results in oxidative stress and DNA damage, leading to upregulation of cytokines and chemokines [7]. This cascade of events induces disruption of skin homeostasis and accelerates the aging process [8,9]. Moreover, the accumulation of damage leads to skin carcinogenesis, inflammatory and pigmentary disorders [10,11,12]. Therefore, aside from photoprotection, elevating the intracellular antioxidant defense system by using antioxidants such as vitamin C and resveratrol is an attractive strategy to mitigate detrimental effects from UVR [13]. However, challenges and potential problems do exist, including stability and penetration [14,15].

Glutathione (GSH), a thiol tripeptide synthesized from three constitutive amino acids—cysteine, glycine and glutamate—plays a prominent role in maintaining tissue homeostasis by preserving cellular redox balance. As a primary intracellular antioxidant, GSH not only protects cells from oxidative damage but also regulates numerous physiological processes, including cell proliferation, apoptosis and immune function [16]. GSH exists in two forms: as reduced monomers acting as hydrogen donors in the detoxification of free radicals and peroxides, and as oxidized dimers (glutathione disulfide [GSSG]) which are produced when GSH is oxidized during various cellular processes. The GSH/GSSG ratio serves as an important indicator of the cellular redox environment, and disruption of this balance can lead to various pathological conditions [17,18]. Further, GSH has been recognized for its skin-lightening property, primarily through its antioxidant effects as well as inhibition of melanogenesis [19]. Environmental stressors like UV exposure result in oxidative stress and consumption of cellular GSH [20]. Maintaining adequate GSH levels therefore bolsters the skin’s natural defense mechanisms and mitigates the adverse effects of oxidative stress, ultimately promoting a more even and radiant complexion [21,22].

**Figure 1:**
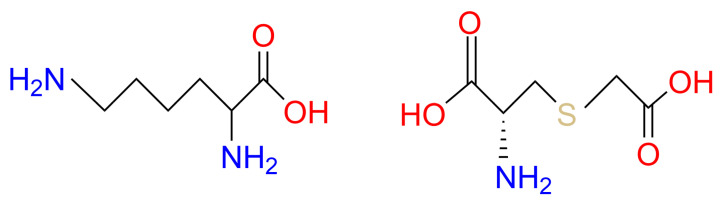
Lysine Carboxymethyl Cysteinate.

GSH has been used commercially for skin lightening in many pharma-cosmeceutical fields. However, cosmetic companies have not used GSH-based ingredients as widely as other antioxidants such as vitamins C and E, probably due to stability and bioavailability challenges [23]. Therefore, the search for novel agents that can elevate GSH levels in the skin has gained significant attention. Our research has long focused on the precursors of GSH biosynthesis, which are effective at replenishing GSH through de novo biosynthesis. On the one hand, we have designed a blend of glutathione amino acid precursors (GAPs) consisting of L-cystine, L-glutamine and glycine. These amino acids can be delivered to skin cells, providing the essential building blocks for de novo synthesis of GSH. GAPs have been found to effectively boost GSH levels in skin and enhance its antioxidant defense, thus improving the ability to cope with stress [23,24]. On the other hand, we have explored synthetic water-soluble sulfur-containing compounds. Lysine carboxymethyl cysteinate (LCC), a lysine salt of carboxymethyl cysteine (Figure 1), has been used as a restructuring agent in hair products to protect skin from heat damage. It is a synthetic substance obtained via lysine salification of S-carboxymethyl-cysteine; therefore, it is L-lysine compounded with S-(carboxymethyl)-L-cysteine (SCC) (1:1). The presence of the carboxymethyl group in LCC is to protect the thiol functional group (S–H) from degradation; lysine is present to increase solubility. Following penetration into the skin, LCC can provide cysteine to produce GSH.

In the present study, the ability of LCC to elevate endogenous GSH levels in primary human keratinocytes and skin tissue samples was demonstrated. This effect was further reinforced by the observation that LCC upregulated key metabolites involved in GSH biosynthesis. Moreover, LCC effectively protected keratinocytes against oxidative stress induced by blue light, and protected skin from UVB-induced damage in a pigmented living skin equivalent (pLSE) model. Beyond antioxidant activity, LCC also suppressed the production of inflammatory mediators, highlighting the multifaceted benefits of LCC for promoting skin health.

## 2. Materials and Methods

### 2.1. Chemicals and Materials

LCC was purchased from Sinerga (HairApp, LCC purity ≥ 98.00%, Gorla Maggiore, Italy, Italy). GSH and N-acetylcysteine were purchased from Sigma-Aldrich. Normal Human Epidermal Keratinocytes (NHEKs), 3D living skin equivalent (LSE, EpiKutis^®^, Guangzhou, China) and pigmented 3D living skin equivalent (pLSE, MelaKutis^®^ Guangzhou, China) were purchased from Guangdong Biocell Biotechnology (Dongguan, China). All the cells used for this study were isolated from heathy Asian skin tissue.

### 2.2. Keratinocyte Treatment and In Vitro GSH, GSSG Quantification

To investigate whether LCC could boost GSH directly, total GSH levels were measured in NHEKs. The cells were seeded in 96-well plates with white walls and clear bottoms. On the following day, cell culture medium was replaced with fresh medium containing 100 μM GSH, or 100 μM N-acetylcysteine (NAC) or 10, 50, and 100 μM LCC. In the non-treatment (NT) group, only the culture medium was refreshed. Then, the cells were cultured for another 24 h and total GSH levels were measured by a luminescence-based assay (GSH-Glo™ Glutathione Assay kit, Promega, Madison, WI, USA).

L-Buthionine sulphoximine (BSO), a specific inhibitor of the de novo GSH synthesis, was used to verify whether LCC was a GSH precursor. The cells were treated with 50 μM LCC or 10 μM BSO, or a combination of 50 μM LCC and 10 μM BSO, on the second day after cell seeding. In the non-treatment (NT) group, only the culture medium was refreshed. The GSH levels were then measured as described above after incubation for 24 h.

### 2.3. ROS (Reactive Oxygen Species) Measurement

In the ROS assay, NHEKs were seeded in a 6-well plate. When cell confluency reached 40–60%, cell culture medium was replaced with medium containing either 10, 50 or 100 μM LCC, or 0.05% vitamin E as a positive control. After incubation for 24 h, the culture medium containing actives was removed and the cells were incubated with DCFH-DA probe (Beyotime Biotechnology, Cat: S0033M, Shanghai, China) for 20 min. The medium containing probes was discarded after incubation and the cells of blue-light and treatment groups were exposed to 10 J/cm^2^ blue light (Chengyue, Cat: cy-bz 120I, Guangzhou, China). The NT group was treated only with culture medium and was not exposed to blue light. ROS production was evaluated 30 min post blue light exposure using flow cytometry (CytoFLEX, Beckman Coulter, Indianapolis, IN, USA).

### 2.4. Detection of LCC Penetration in Epidermis via Fluorescein Isothiocyanate (FITC) Labeling

**Figure 2:**
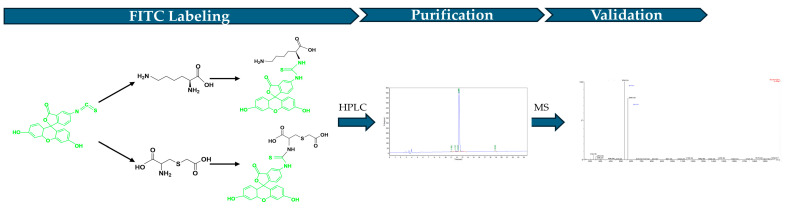
Workflow of FITC-labeled LCC.

The FITC (Yunhui Biotech, Cat: ZB23145, Zibo, Shandong, China) labeled LCC was generated by conjugating FITC with the N terminal of the peptide (Figure 2). To produce the FITC-labeled LCC, 200 mg LCC diluted in PBS was mixed with 200 mg FITC dissolved in DMSO. Then, the mixture was added into N, N-Diisopropylethylamine (DIEA, Jiuding Technology, Cat: N003A-100mL, Shanghai, China) and incubated for 4 to 6 h to proceed the reaction. After fully reacting, the final product was pumped into the HPLC (Shimadzu, LC-2010, Fukuoka, Japan) to purify FITC-labeled LCC. After purification, the final product structure was verified by mass spectrometry (Waters, ZQ 2000 LC/MS System, Milford, MA, USA).

The LSE model was treated with 25 μL 0.1% FITC-labeled LCC topically, and incubated at 37 °C. The culture medium and LSE models were collected at 2 h, 4 h, 8 h and 24 h after treatment. The fluorescence density in the medium was measured by BioTek Epoch (Agilent Technology, Santa Clara, CA, USA). The collected LSE models were fixed in cold 4% neutral buffered formalin solution (SIGMA-ALDRICH, Co., Cat. 252549, Saint Louis, MO, USA), dehydrated, and embedded in paraffin. The fixed model was then sectioned into 5 μM slides and the image was acquired using a microscope (Olympus, BX43, Center Valley, PA, USA).

### 2.5. Real-Time Quantitative Polymerase Chain Reaction (qPCR) of GSH-Associated Genes in the LSE Model

To evaluate if topical application of LCC could recover GSH function, the LSE model was utilized and the expression of GSH-associated genes analyzed. LCC at 0.27% was applied on top of the LSE model on Day 3 and Day 5 after the test was initiated. Only the culture medium was refreshed in the NT group. The models were harvested after incubation for 24 h.

Total RNA in collected LSE models was extracted with RNAiso Plus (TaKaRa, Cat: 9108; Cat: RR036). RNA concentration was then measured with a Nanodrop spectrometer (Thermo Fisher Scientific, Waltham, MA, USA) and RNA was reverse transcribed to cDNA with PrimeScript II 1st Strand cDNA synthesis kit according to manufacturer’s instructions. qPCR was performed with SYBR^®^ Premix Ex Taq™ on the ABI Vii 7 Real-Time PCR System (Applied Biosystems, Thermo fisher scientific, Carlsbad, CA, USA) with primers of *GCLC* (F:5′-GCATTATTGACGAACTGGCTACA-3′; R:5′-CTTAATCAATTTCTGGCTCACTGG-3′) and *GSTA1* (F:5′-AGTGTTGATTGTGCCTGTTGTG-3′; R:5′-ACTAAGTCAGCGAATAGGAGTTGT-3′). *ACTB* was selected as the housekeeping gene. The fold changes of gene expression were calculated with (2^(−ΔΔCt)^) relative to the NT group.

### 2.6. Spatial Metabolomic Analysis

**Figure 3:**
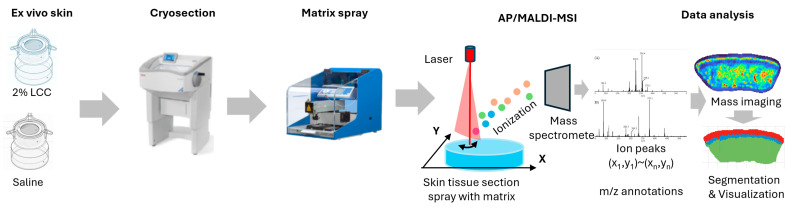
Workflow of AP/MALDI based spatial metabolome analysis (the color represents the total ions intensity).

The ex vivo skin models were obtained from surgery discards—with written consent—by the Archgene company (Shanghai, China). The skin explants were disinfected overnight at 4 °C and pre-treated with scissors to get rid of excess fat tissue. Then, the skin was punched to 6 mm skin tissue and cultured in a 12-well insert plate with culture medium at 37 °C under a humidified atmosphere of 95% air and 5% CO_2_. Explants were then topically treated with 2% LCC or saline for 24 h, after which they were embedded in 10% gelatin and frozen with liquid nitrogen. Frozen samples were shipped to PANOMIX (Suzhou, China) for spatial metabolome analysis. An AP/MALDI UHR ion source (MassTech, Columbia, MD, USA) was coupled with a Thermo QE Plus mass spectrometer (Thermo Fisher Scientific, Waltham, MA, USA) for all data acquisition. During full scans, a mass range of *m*/*z* 120–1300 was used with a resolution of 35,000. High-resolution mass spectrometry imaging (MSI) image reconstruction and visualization were carried out using MSI-processing algorithms and a standalone software version of the biodeep platform (http://www.biodeep.cn; assessed on 6 February 2025) (Figure 3).

### 2.7. UVB-Challenged pLSE Model

The pLSE models in the UV and LCC groups were treated with 50 mJ/cm^2^ UVB exposure once daily from Day 1 to Day 6. The LCC group was also treated with 5 µL 0.1% LCC on the surface of pLSE models on Days 2, 4 and 6 after UVB exposure. The NT group was not exposed to UVB, and only the culture medium was changed. On Day 7, all models and culture media were collected for further analysis.

### 2.8. L* Value, Melanin Content Measurement and Distribution Analysis

L* value is a common parameter used to indicate the brightness of skin. After sample collection, the L* value of each pLSE model was determined with Chroma meter (Cortex Technology, DSM II, Nordjylland, Hadsund, Denmark). The total melanin content of each sample was then measured. The pLSE model of each group was lysed with 1 mL of 1 M NaOH containing 10% DMSO and incubated in an 80 °C water bath for 40 min. After incubation, 200 μL of the supernatant was applied to a 96-well plate and the melanin content was measured at OD405 using a microplate reader. The melanin distribution in the pLSE model was evaluated with Masson–Fontana melanin staining kit (Yike Biotechnology Service Co., Ltd., Cat: YK2318, Xi’an, China) according to the manufacturer’s instructions, as described previously [25].

### 2.9. Immunofluorescence Staining

Tissue sections of the pLSE model were prepared as described above in Section 2.7. Filaggrin was stained with Anti-Filaggrin antibody (Abcam, Cat: ab218397, Waltham, MA, USA) according to manufacturer’s instructions and nuclei were stained with Hochester. The images were acquired using a microscope (Olympus, BX43, Center Valley, PA, USA) and analyzed with Image Pro Plus (Media Cybernetics Inc., Rockville, MD, USA).

### 2.10. Cytokine Measurement

Culture supernatants of the pLSE model were collected to analyze cytokine levels with Enzyme-Linked Immunosorbent Assay (ELISA). IL-1α was detected using Human IL-1 alpha ELISA Kit (Abcam, ab100560, Waltham, MA, USA) and PGE2 was detected using Prostaglandin E2 ELISA Kit (Abcam, ab133021, Waltham, MA, USA) following the manufacturer’s instructions.

### 2.11. Statistical Analysis

The in vitro results in the figures are presented in the form of mean ± standard deviation (SD). Statistical significance was calculated using a T-test with two tails and equal SD (*p* < 0.05 was considered statistically significant).

## 3. Results

### 3.1. LCC Elevates GSH Level in Skin Keratinocytes

**Figure 4:**
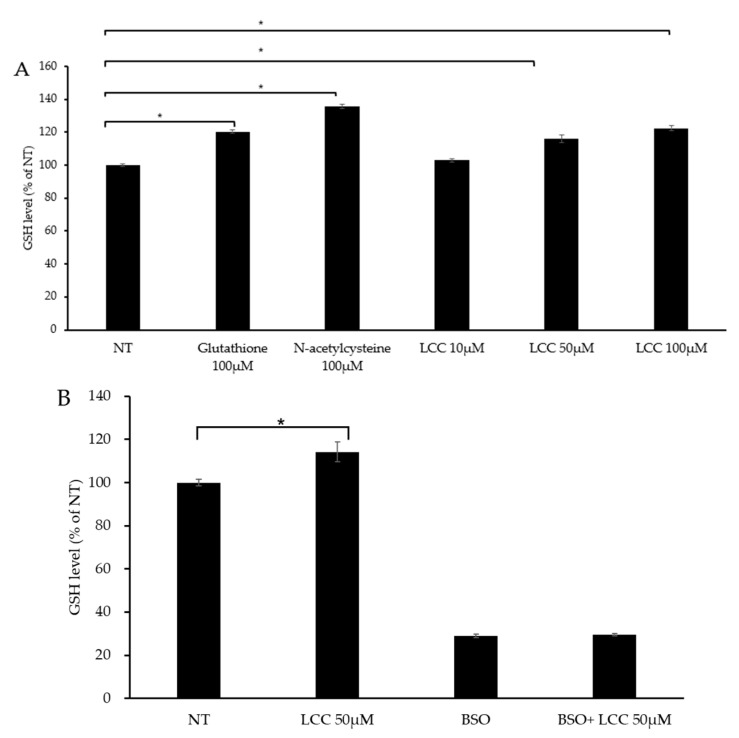
GSH boosting effect of LCC in NHEKs. (**A**) Effects of GSH, NAC and LCC on GSH levels in NHEKs. (**B**) Effects of LCC and/or BSO on GSH production in NHEKs. All values are mean ± SD (n = 3). * *p* < 0.05 between groups.

To evaluate LCC’s capability in boosting skin GSH level, human keratinocytes were treated with different concentrations of LCC, along with GSH and NAC as references. After 24 h of treatment, GSH level was significantly increased by GSH and NAC, as expected (Figure 4A). The change in GSH level under LCC treatment showed a dose-dependent pattern. A dose of 10 μM LCC elevated GSH slightly, by 3%; however, 50 μM and 100 μM LCC increased GSH levels significantly, by 16% and 23%, respectively (Figure 4A).

Glutamate cysteine ligase (GCL) is the rate-limiting enzyme in the GSH biosynthesis pathway. GCL catalyzes cysteine and glutamine to form the dipeptide γGluCys. BSO is a specific inhibitor of GCL which can deplete cellular GSH and trigger oxidative stress [26]. To investigate whether LCC increased GSH level through GSH synthesis, BSO was co-treated with LCC. The GSH level was significantly elevated by 50 μM LCC, and BSO suppressed GSH levels, as expected. When BSO was co-treated with LCC, the effect of LCC was compromised (Figure 4B). This result indicated that LCC boosted GSH through increasing GSH synthesis.

### 3.2. LCC Protects Skin Keratinocytes from Oxidative Stress

**Figure 5:**
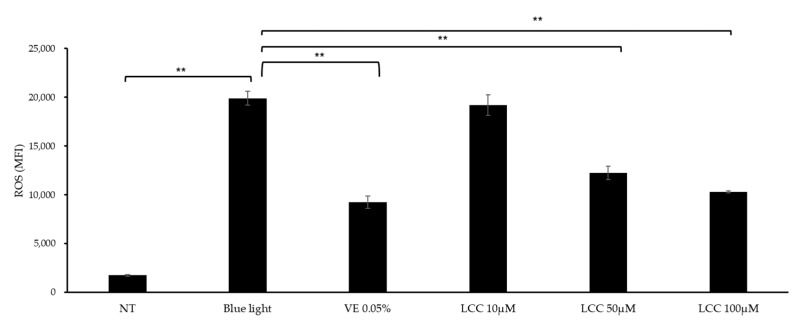
ROS inhibition effect of LCC in blue-light-exposed NHEKs. All values are mean ± SD (n = 3). ** *p* < 0.01 between groups.

ROS inhibition efficacy was evaluated in blue-light-exposed NHEKs. As shown in Figure 5, blue light exposure exaggerated ROS levels by 10 times, compared with the NT group. At a low concentration (10 μM), LCC lowered ROS generation by 3%. At higher dosages, the ROS inhibition function was more manifestly evident. Doses of 50 μM and 100 μM LCC decreased ROS levels by 38% and 48%, respectively.

### 3.3. Permeation of Skin by LCC in the LSE Model

**Figure 6:**
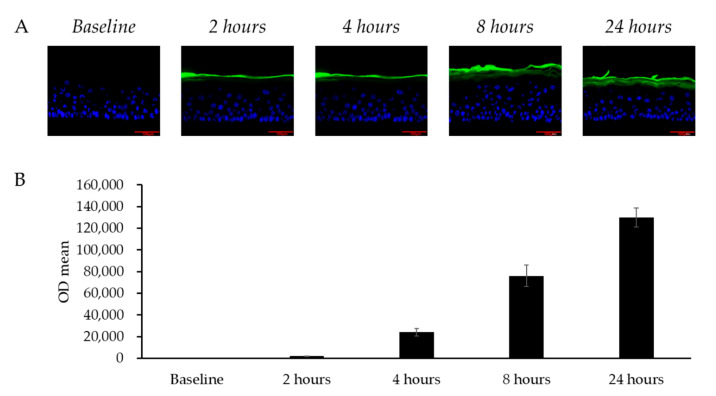
Transdermal penetration of skin by LCC in the LSE model. (**A**) Fluorescence microscopy images of vertical sections of LSE models treated with FITC-labeled LCC (green); scale bar = 100 μm. Nuclei are stained with DAPI (blue). (**B**) FITC intensity in the culture medium from baseline to 24 h. All values are mean ± SD (n = 3).

To evaluate whether topically applied LCC could diffuse into skin, FITC-labeled LCC was applied on the LSE model and the fluorescence of LCC was analyzed. Figure 6 shows how, in the 24 h after application, LCC gradually spread from the stratum corneum layer towards the granular layer. The fluorescence in the culture medium indicated that the LCC sample permeated through the model and remained in the culture medium. Consistent with the fluorescence distribution in the images, the fluorescence density in the medium accumulated over 24 h (Figure 6B), indicating that topical treatment of LCC passed through the LSE model gradually.

### 3.4. Topical LCC Regulates Glutathione Metabolism Genes

As mentioned in Section 3.1, GCL is a critical enzyme in the GSH synthesis pathway. GCL is composed of catalytic (GCLc, encoded by the *GCLC* gene) and modifier (GCLm) subunits. Therefore, the expression of *GCLC* was selected to investigate the function of LCC in GSH synthesis. The glutathione-S-transferases (GSTs) are a large superfamily of ubiquitous detoxification enzymes involved in cellular detoxification function of GSH. GSTA1 is a cytosolic protein belonging to the GST family [27]. Thus, *GSTA1* gene expression was measured to demonstrate the detoxification function of LCC.

**Figure 7:**
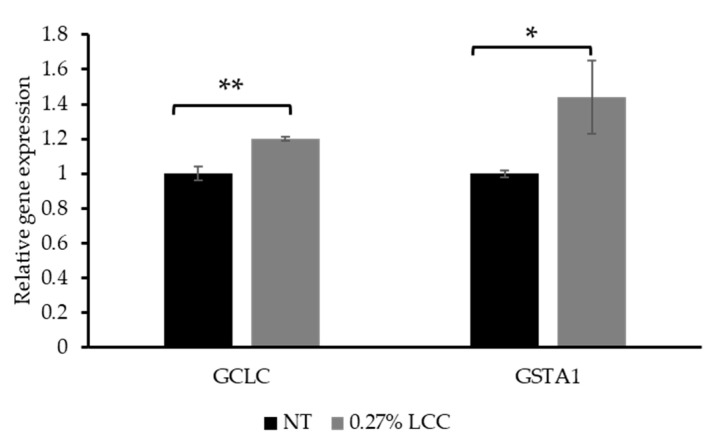
Effect of topical LCC treatment on the expression of genes associated with GSH synthesis and detoxification functions in LSE models. The relative expression levels of *GCLC* and *GSTA1* were quantified via qPCR. All values are mean ± SD (n = 3). * *p* < 0.05 versus the NT group; ** *p* < 0.01 versus the NT group.

As shown in Figure 7, topical application of 0.27% LCC significantly increased *GCLC* and *GSTA1* expression. This result suggested that LCC improved the cell`s ability to synthesize and utilize GSH, thereby protecting skin against oxidative damage and enhancing detoxification functions.

### 3.5. Topical LCC Increases GSH Signal in the Epidermis Region

To further validate if topical LCC application could increase skin GSH, and to explore the impact of LCC treatment on skin metabolism, we conducted high-resolution (20 μm) spatial metabolomic analysis of human ex vivo skin samples treated with 2% LCC or saline using atmospheric pressure matrix-assisted laser desorption/ionization mass imaging (AP/MALDI-MSI) [28].

**Figure 8:**
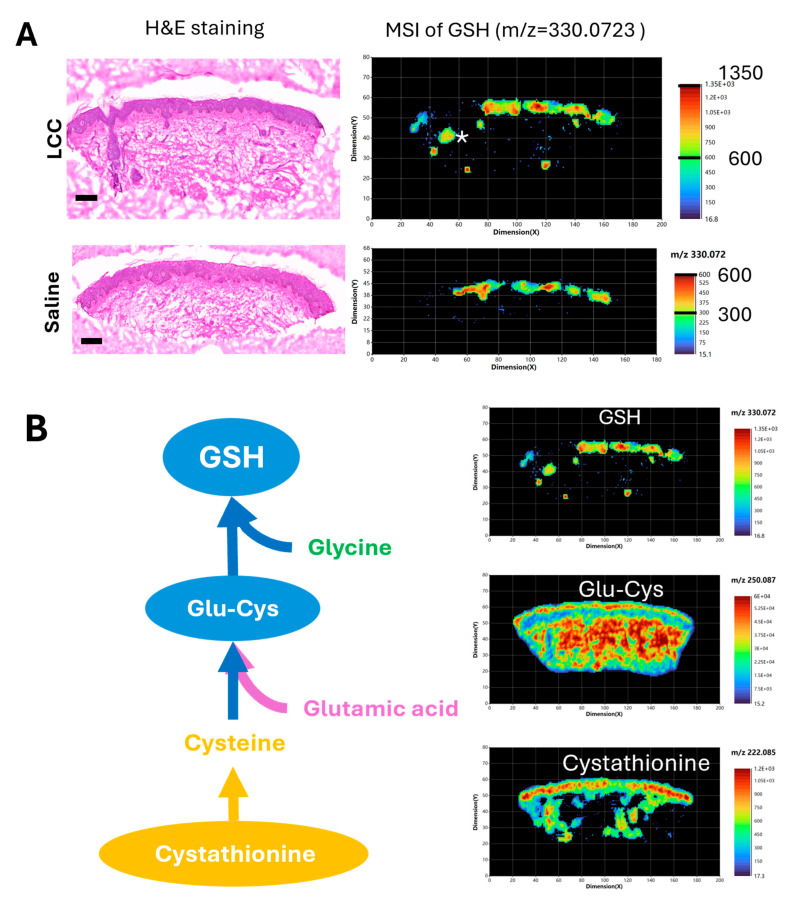
Spatial metabolomic analysis of ex vivo skin samples. (**A**) Hematoxylin and eosin (H&E) staining and representative MSI of GSH (m/z 330.0732) using positive ion mode. Scale bars in H&E images: 200 μm. * in the MSI image of LCC treated skin represents a hair follicle. (**B**) GSH de novo biosynthesis pathway and representative MSI of metabolites in skin sample treated with 2% LCC. Glu-Cys—L-gamma-glutamyl-L-cysteine.

We identified that the peak at *m*/*z* 330.0732 matched to the GSH ion ([M+Na]+) with a mass error of 5 ppm (part per million). This GSH signal was mainly located in the epidermis region of skin samples, as well as in the skin follicle of the LCC-treated sample (Figure 8A). The GSH signals in epidermis regions were generally higher in the sample treated with LCC than that treated with saline, supporting the GSH boosting effect of LCC. Next, we visualized metabolites involved in GSH biosynthesis in the LCC-treated skin samples (Figure 8B). Cystathionine was identified with a peak at *m*/*z* 222.0851, showing a higher distribution in the epidermis, and was also present in the dermis part with a non-uniform distribution. Glu-Cys was identified with a peak at *m*/*z* 250.087, showing a more even distribution in skin than GSH or cystathionine. Glu-Cys signals were higher in the epidermis region and the deep reticular region than in the superficial papillary region in the dermis.

### 3.6. Topical LCC Protects Skin from UV Damage

**Figure 9:**
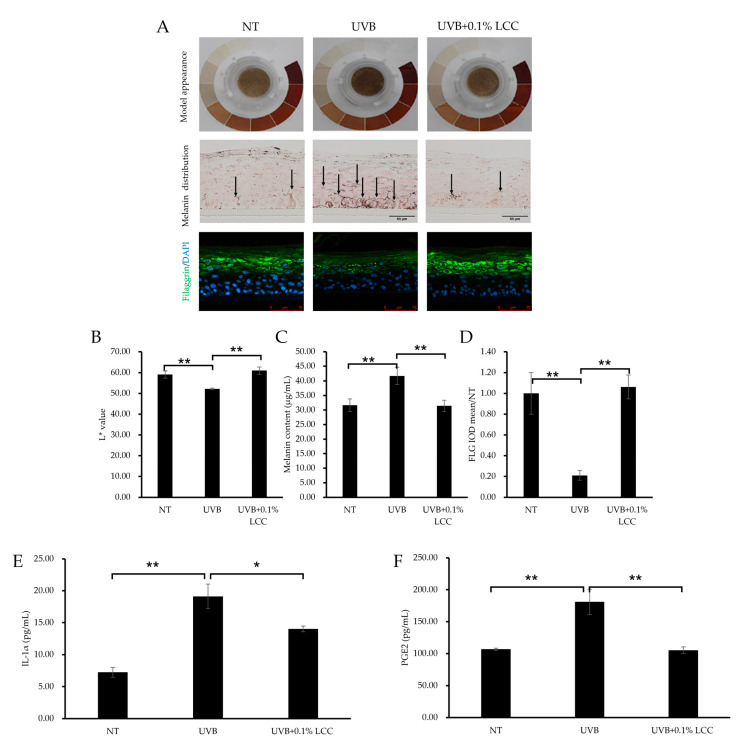
UV protection effect of LCC in the pLSE model. (**A**) Model appearance, melanin distribution (scale bar = 50 μm) and filaggrin (scale bar = 75 μm) immunofluorescence staining of the pLSE model. Arrows indicate the melanin pigment. (**B**–**D**) The quantification of L* value, total melanin content and filaggrin fluorescence intensity of the pLSE, respectively. (**E**,**F**) The IL-1α and PGE2 levels in the supernatants from the pLSE model. All values are mean ± SD (n = 3). * *p* < 0.05 between groups; ** *p* < 0.01 between groups.

UV exposure can cause changes in skin tone and inflammation. To investigate the UV protection efficacy of LCC, the pLSE model was exposed to UVB. After UVB exposure, the model exhibited a darker tone as compared with the NT group, which was further evidenced by a 12% reduction in the L* value (Figure 9A(upper),B). Consistent with the change of tone, melanin was also accumulated in the model (Figure 9A) and the total melanin content was increased by 32% (Figure 9C). LCC application significantly lightened the tone of the pLSE model and improved the L* value by 17%. Accordingly, the melanin deposition in the pLSE model was reduced (Figure 9A middle) and the total melanin content declined by 25% (Figure 9C).

UVB also caused skin barrier damage. As shown in Figure 9A,D, after UVB exposure, filaggrin was dramatically reduced, by 79%. Treatment with 0.1% LCC increased the level of filaggrin by 404%, restoring its expression to a level comparable with NT (Figure 9D). Additionally, inflammatory cytokine releases were induced by UVB exposure. The IL-1α level increased by 163% (Figure 9E) and the PGE2 level increased by 69% (Figure 9F). LCC suppressed levels of IL-1α and PGE2 by 27% and 42%, respectively.

These results demonstrated that LCC provided protection for the skin against UVB by reversing changes in skin tone, melanogenesis, barrier interruptions and inflammatory responses induced by UVB exposure.

## 4. Discussion

- The realm of skincare has witnessed significant advancements in recent years; however, topically applied ingredients face challenges related to penetration and stability. Prodrugs, which are biologically inactive compounds strategically designed to convert into active therapeutics within the body, are of immense importance in medicine by virtue of their abilities to enhance drug efficacy, safety and delivery [29,30]. In contrast, the potential of prodrug technology remains largely unexplored in the cosmetic industry. We have been working on the development of precursor technologies which are essential building blocks in the de novo synthesis of important biomolecules, restoring their reserves and thereby enhancing skin health. One example is the GSH precursor blend GAP, which can stimulate the endogenous, intracellular production of GSH, enhancing the skin’s natural defense mechanisms against environmental insults [23,24]. The incorporation of GAP into skincare formulations underscores this advancement in skincare science, as it offers a targeted and effective approach for promoting cellular resilience and rejuvenating skin.
- GSH is the most prominent non-protein antioxidant. It plays a crucial role in maintaining cellular integrity, and contributes to longevity by mitigating oxidative stress-induced damage [16,31,32]. Numerous studies have emphasized the advantages of boosting GSH levels in the skin, leading to a surge in discoveries of active ingredients to replenish GSH stores depleted by external stressors [33,34]. Following our prior work on GAP in which the body’s own synthesis of GSH was encouraged, we present in this paper the findings of a study examining the impact of a newly discovered compound, LCC, on skin GSH content. We carried out this work because LCC has been hypothesized to boost GSH through a slightly different mechanism to that of GAP. The availability of cysteine is among the major determinants of GSH synthesis. LCC consists of SCC which stabilizes the reactive thiol group within cysteine, thereby ensuring a consistent supply of this essential amino acid for GSH synthesis. This mechanism positions LCC as a powerful agent for enhancing intracellular GSH levels. Our findings revealed that LCC significantly elevated GSH levels in 2D cell cultures (Figure 4), as well as in human skin tissues (Figure 8). LCC effectively penetrated the epidermis where it played a role modulating glutathione metabolism (Figure 6 and Figure 8). Additionally, using a Franz diffusion cell system, we demonstrated that LCC can penetrate the skin from a cosmetic leave-on formulation (Tables S1 and S2). Moreover, LCC exhibited promising protection capabilities under various stress conditions, including exposure to blue light and UVB (Figure 5 and Figure 9), highlighting the potential of LCC as a novel and effective strategy to improve the skin’s endogenous defense system.
- It was shown in keratinocytes that the upregulation of GSH following LCC treatment was abrogated in the presence of BSO, preventing the formation of GSH by blocking the first step of its synthesis (Figure 4B) [26]. This suggested that the mechanisms for GSH regulation by LCC were through the de novo GSH synthesis pathway. Also, LCC upregulated GSH metabolism genes including *GCLC* and *GSTA1* in LSE models (Figure 7), suggesting that an overall ability to synthesize and utilize GSH was improved by LCC. Data from spatial metabolomic analysis showing that LCC increased the levels of key metabolites involved in GSH biosynthesis further substantiated this finding (Figure 8).
- The data obtained from penetration studies of skin 3D models, as well as from spatial metabolomic analysis, provided compelling evidence that the great majority of LCC’s action was exerted in the epidermis (Figure 6 and Figure 8), highlighting its crucial role in supporting epidermal health. As expected, LCC inhibited ROS production in keratinocytes exposed to blue light, a widely used inducer of oxidative stress (Figure 5). Mechanistically, this protection effect could stem from GSH replenishment, given that that GSH directly scavenges ROS [35]. Indeed, the GSH/GSSG ratio in the UV-exposed skin area in vivo was discovered to be sharply reduced after UV exposure, compared with the control area [23]. Therefore, by counteracting GSH depletion induced by environmental stressors, LCC likely restored cellular GSH reservoir, and thus reduced oxidative damage to macromolecules.
- GSH regulates melanogenesis through reducing intracellular tyrosinase activity and inhibiting melanin synthesis. In the melanocytes, LCC significantly reduced melanin content (Figure S1). In addition to enhancing antioxidant defenses, topically applied LCC conferred multiple advantages to the epidermis, including barrier protection, suppression of inflammation and improvement in skin tone, as demonstrated in pLSE models subjected to UVB challenge (Figure 9). We found in this model that LCC markedly suppressed PGE2 and IL-1α expression, two major proinflammatory cytokines mediating photodamage [36,37,38]. In line with this, our study in the LSE model showed that topical application of 0.27% LCC did not induce inflammatory infiltration, compared with untreated controls (Figure S2). Oxidative stress and inflammation are interdependent processes that can exacerbate each other, contributing to skin photodamage [39]. It is logical to infer that the potent antioxidant activity of LCC might play a role in the mitigation of inflammatory responses. Notably, the inflammatory response is coordinated by a large range of cellular and molecular mediators that form complex regulatory networks. For instance, UV-induced tissue dysfunction in the skin has long been considered to derive in part from UV-induced senescent cells, among which the senescence-associated secretory phenotype (SASP) promotes chronic inflammation, and even tumorigenesis [40]. Whether LCC suppresses cytokine production through the regulation of keratinocyte senescence or through other specific signaling pathways warrants further investigation. In addition, the employment of more advanced and complicated in vitro models that incorporate other cell types such as immune cells would greatly facilitate this line of research.
- As for the depigmentation effect, the reduction in melanin and increase in L* may arise from GSH’s skin-lightening activity, as demonstrated in clinical studies with topically applied GSH [41]. However, additional mechanisms of action could contribute to the observed protection. Inflammation has been recognized as a causal factor in exacerbating melanogenesis [42], and LCC’s potential to suppress pro-inflammatory mediators might indirectly disrupt pigmentation pathways. For example, melanocytes express PGE2 receptors which react to the PGE2 released by keratinocytes, facilitating the transfer of melanosomes to surrounding keratinocytes [43]. We speculate that LCC could reduce PGE2 production by keratinocytes, and further prevent this melanosome transfer. Further studies are necessary to delineate the relative contributions of GSH-boosting and anti-inflammatory activities, so as to clarify the efficacy of LCC in mitigating UV-induced hyperpigmentation.
- The demonstrated efficacy of LCC in mitigating UV-induced damage in LSE models underscores its photoprotection potential. These findings highlight the limitations of conventional sunscreens, which primarily block UV radiation but incompletely prevent sun-induced ROS formation and downstream cellular and tissue damage [44,45]. The accumulation such damage, particularly DNA damage and oxidative stress, not only accelerates the skin aging process, but also promotes skin carcinogenesis through mechanisms involving gene mutations, impaired DNA repair, and chronic inflammation [46,47]. Therefore, complementing conventional sunscreens with ingredients possessing antioxidant properties like LCC can be considered as a novel application in skincare solutions which may synergistically minimize photodamage and maximize photoprotection. This aligns with emerging studies of sunscreens incorporated with ferulic acid, nicotinamide or rosmarinic acid [48,49,50]. However, few clinical studies have yet been conducted, and the development of sunscreen formulations containing antioxidants that preserve the effectiveness of both could be challenging.
- In conclusion, the above findings pave the way for future studies to explore the possible applications of LCC as a key ingredient in addressing skin conditions associated with compromised epidermal function, by virtue of its antioxidant power and its ability to resist environmentally induced oxinflammation. Moving forward, the adoption of multi-omics approaches, including transcriptomics, proteomics, microbiome and metabolomics, will be instrumental in unraveling the intricate mechanisms of multidirectional actions of LCC from a holistic perspective.

## 5. Conclusions

In conclusion, the present study conducted an extensive examination of the various biological effects of LCC. LCC exhibited significant potential as a GSH precursor, particularly through its antioxidative properties and ability to boost GSH levels. Further research revealed the skin brightening and UV-protective effects of LCC in vitro, suggesting that LCC has the potential to be developed into a key ingredient for preserving skin integrity and delaying skin photoaging.

## Data Availability

The data of this study are available from the corresponding author upon reasonable request with permission of the Unilever Company.

## Supplementary Materials

The following supporting information can be downloaded at https://www.mdpi.com/article/10.3390/antiox14050606/s1, Table S1: Dermal delivery of LCC in a nano emulsion ex vivo; Table S2: The formulation sheet of LCC in the nano emulsion; Figure S1: Effect of LCC on melanin production; Figure S2: The effect of LCC on the 3D skin equipment model. (a) H&E, the scale bar equals 50 μm. (b) Expression of IL-1α in 3D skin equivalent model.
