# Supplementary material for: Lysine Carboxymethyl Cysteinate, as a Topical Glutathione Precursor, Protects Against Oxidative Stress and UVB Radiation-Induced Skin Damage
Source: Antioxidants (Basel). 2025 May 17;14(5):606. doi: 10.3390/antiox14050606 (PMC12108675; doi:10.3390/antiox14050606)
Supplement: Supplementary file 1 [file antioxidants-14-00606-s001.zip › Antioxidants- 3587137- supplementary.pdf]

## Supplementary materials

## Lysine Carboxymethyl Cysteinate, as a topical glutathione precursor, protects against oxidative stress and UVB radiation-induced skin damage

Materilas and Methods

Dermal delivery study on LCC in a nano-emulsion. Transdermal flux was measured over a period of up to 24 hours after application of the LCC prototype to cadaver skin. At the end of the 24 hours time period, the skin was tape-stripped eight times. The tape strips were collected and binned together as tape strips 1-3 and tape strips 4-8. The skin was then split into the epidermal and dermal compartments. The LCC was then extracted from the tape strips and from the skin compartments.
Donor ID#: RW011522; Sex: Male; Age: 56; Site: Right Posterior Leg; Supplier: New York Firefighter’s Skin Bank

Results

Supplementary table (S1) dermal delivery of LCC in a nano emulsion ex vivo

| | **Total ug/cm$^2$±SEM** | **Percent of dose** | **Micromolar** |
|---|---|---|---|
| Tape strip 1-3 | 16.67±3.13 | 52.24 | |
| Tape strip 4-8 | 11.44±2.53 | 35.86 | |
| **Total stratum corneum** | **28.11±4.17** | **88.12** | |
| Epidermis | 1.56±0.30 | 4.88 | 2068 |
| Dermis | 0.45±0.04 | 1.41 | 97 |
| Receptor fluid 10 | 0.04±0.0 | 0.11 | |
| Receptor fluid 24 | 0.001±0.01 | 0.03 | |
| Mass balance | | 94.53 | |

Supplementary table (S2) The formulation sheet of LCC in the nano emulsion

| **Component** | **w/w %** |
|---|---|
| Deionized water | 53.95 |
| NaOH | 0.2 |
| NaCl | 1.6 |
| EDTA | 0.3 |
| Glydant plus liquid | 0.1 |
| LCC | 1.35 |
| Caprylic/capric triglyceride | 36.7 |
| DC ES-5300 | 4.6 |
| KSG210 | 1.2 |
| TOTAL | 100 |

Materilas and Methods

Normal Human Epidermal Melanocytes (NHEMs, Lot: MC24112603, Guangdong Biocell Biotechnology, Guangzhou, China) were seeded in 6-well plates. When the cell confluency reached 40-50%, the cells were treated with 50μM LCC and incubated for 72 hours. After incubation, the culture medium was removed and the cells were washed with PBS twice. Then the melanocytes were collected with trypsin and moved into centrifuge tubes. The colleced celled were lysed with 1mL lysis buffer of 1mol/L NaOH containing 10%DMSO and incubated water bath at 80°C for 40 minutes. Then, 200μL cell lysates were transferred to a new 96-well plate, and absorbance was measured at 405nm using BioTek Epoch (Agilent Technologies, Inc, Santa Clara, CA, US). Serial dilutions of pure melanin were prepared as references. Melanin content was calculated using a melanin standard curve.

Results

The result indicaed that LCC signicantly reduced melanin content in the melanocytes.

Supplementary figure (S1)

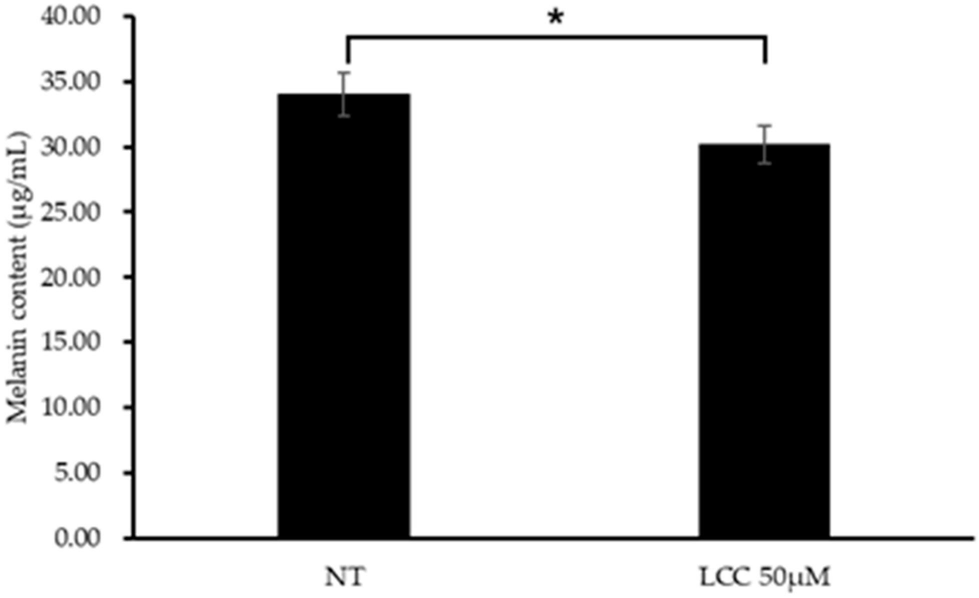


Figure S1: Effect of LCC on melanin production. All values are mean±SD (n=3). * $p<0.05$ between groups.

Materilas and Methods

3D living skin equivalent model (LSE, EpiKutis®, Lot: ES230808, Guangdong Biocell Biotechnology, Guangzhou, China) were utilized to determine the histolgical changes and potential skin irriation risk. In the NT group, only the culture medium was refreshed daily. In the LCC group, the models were treated topically with $2mg/cm^2$ of 0.27% LCC for four days. After the last application, all EpiKutis® were cultured for another 24 hours and collected for futher analysis.

The collected LSE models were immediately fixed in cold 4% neutral buffered formalin solution (SIGMA-ALDRICH, Co., Cat. 252549, Saint Louis, MO, USA), dehydrated, and embedded in paraffin. Then, the tissues were sliced into 5 μm vertical sections. The sections were further deparaffinized and rehydrated through graded ethanol series and stained with hematoxylin and eosin (H&E) (Beyotime Biotechnology, Cat: C0107 and C01055, Shanghai, China) for histological evaluation. The collected culture medium was measured for IL-1$\alpha$ with ELISA kit (Abcam, ab100560, Waltham, MA, USA).

Results

LCC did not show a significant alteration in IL-1$\alpha$ level as comapred to NT group. Supplementary figure (S2)

(a)

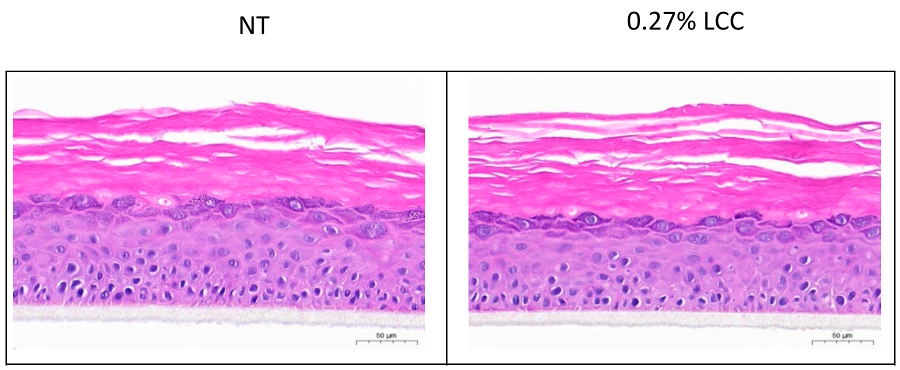


(b)

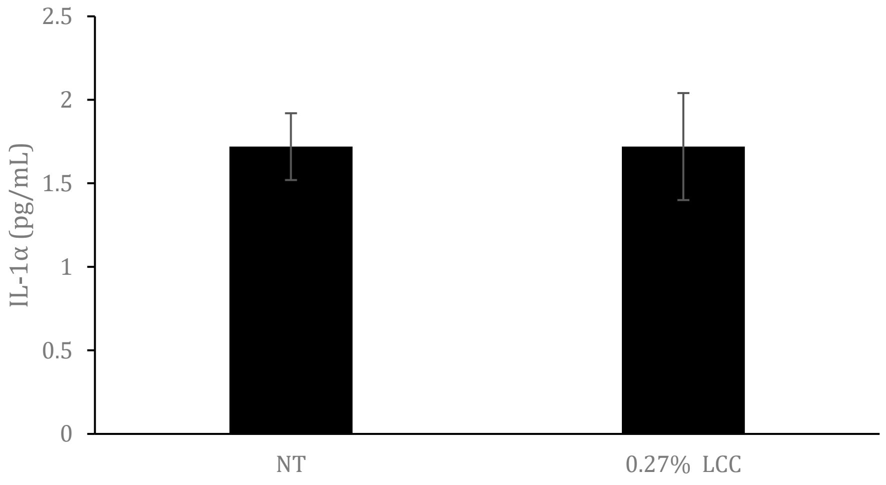


Figure S2: The effect of LCC on the 3D skin equivment model. (a): H&E, the scale bar equals 50 μm. (b): Expression of IL-1$\alpha$ in 3D skin equivalent model. All values are mean±SD (n=3).
